# The University of Queensland study of physical and psychological outcomes for claimants with minor and moderate injuries following a road traffic crash (UQ SuPPORT): design and methods

**DOI:** 10.3402/ejpt.v5.22612

**Published:** 2014-05-02

**Authors:** Justin Kenardy, Michelle Heron-Delaney, Nicholas Bellamy, Michele Sterling, Luke Connelly

**Affiliations:** 1 Centre of National Research on Disability and Rehabilitation Medicine (CONROD), School of Medicine, RBWH, The University of Queensland, Brisbane, QLD, Australia; 2 School of Psychology, Australian Catholic University, Brisbane, QLD, Australia; 3 Australian Centre for Economic Research on Health, School of Economics, RBWH, The University of Queensland, Brisbane, QLD, Australia

**Keywords:** Posttraumatic stress, motor vehicle crash, longitudinal, physical recovery, minor injuries

## Abstract

**Background:**

To date research investigating how mental health impacts physical recovery following a road traffic crash (RTC) has focused on cohorts with severe injuries. The UQ SuPPORT study aims to study the physical and psychological outcomes of claimants with minor injuries following an RTC under the Queensland common law compulsory insurance scheme.

**Objectives:**

This paper outlines the protocols of this study as a platform for future publications.

**Methods:**

The 2-year longitudinal cohort study collected interview and survey data from claimants at 6, 12, and 24 months post-RTC. Measures used in the telephone interview included the DSM-IV Composite International Diagnostic Interview for posttraumatic stress disorder, generalized anxiety disorder, major depressive episode, panic attacks, agoraphobia; and self-reported disability (WHO-DAS-II). Quality of life (SF-36v2), alcohol use (AUDIT), social support (MSPSS), quality-adjusted life years (EQ-5D), and return to work outcomes were assessed via postal questionnaires.

**Results:**

A total of 382 claimants consented to participate at the beginning of the study, and these participants were approached at each wave. Retention was high (65%). The average age of participants at Wave 1 was 48.6 years, with 65% of the sample sustaining minor injuries (Injury Severity Score=1–3).

**Conclusions:**

This study has collected a unique sample of data to investigate recovery patterns of claimants with minor injuries. Future publications will more fully assess the effects of the collected measures on recovery rates 2 years post-RTC.

Worldwide, up to 50 million people suffer a non-fatal injury from a road traffic crash (RTC) each year, leading to long-term disability in many individuals (World Health Organization, 2009). Over the past decade, there has been much research assessing both the physical and psychological consequences of RTCs. Research shows that physical consequences of RTCs are significant and ongoing (Ruseckaite, Gabbe, Vogel, & Collie, 2012), and even minor RTC injuries evoke long-term health problems (e.g., Mayou & Bryant, 2001; Smith, Mackenzie-Ross, & Scragg, 2007). The World Health Organization (2009) has estimated that road traffic injuries will rise to be the third leading cause of disability-adjusted life years lost (DALYs) by 2020. RTCs clearly evoke long-term physical consequences, and Australia reports spending a considerable 3.6% of its GDP on RTCs (Bureau of Transport and Regional Economics, 2000). There has been an increased focus on psychological morbidity following RTCs (Ehlers, Mayou, & Bryant, 1998), with poor mental health having important consequences in terms of quality of life, absenteeism from work, and higher levels of pain and disability in RTC survivors (Beesdo et al., 2010; Matthews, 2005). It has become increasingly apparent that physical functioning/pain and mental health interact to produce long-term health outcomes (Sterling & Kenardy, 2011). While the majority of individuals will recover from RTCs, a significant minority will experience mental health problems and/or pain/disability, which require identification and treatment to stop the development of long-term psychiatric disorders and/or chronic pain (Mayou & Bryant, 2002).

The principal psychological disorders, which are often diagnosed following an RTC, are posttraumatic stress disorder (PTSD), depression, driving phobias, and other anxiety disorders (Mayou & Bryant, 2002). The prevalence of PTSD following an RTC varies considerably across studies, ranging from 6 to 45% (e.g., Matsuoka et al., 2008; Mayou & Bryant, 2002; Ursano et al., 1999). Prevalence estimates of other psychological disorders have not been reported in RTC samples; however, data from self-reported symptom questionnaires indicate prevalence estimates of depressive symptoms to be 10% (Ehring, Ehlers, & Glucksman, 2008), anxiety symptoms to be 36% (Smith et al., 2007), and travel phobia to be 20% (Ehring et al., 2008) in RTC samples. The general Australian population estimate for depression is 4% and for anxiety-related disorders is 8% (Australian Bureau of Statistics, 2008). The prevalence of mental health issues is clearly elevated in populations who are injured in RTCs.

The vast majority of research to date has focused on RTC survivors who were hospitalized and suffered serious or life-threatening injuries. Very little is known about the psychological and physical outcomes for RTC survivors with minor injuries. This represents an important but neglected group, as the few studies which have assessed psychological morbidity in RTC survivors with minor injuries reveal that if left untreated, psychological problems are prevalent and can continue over an extended period of time (UK: Ehlers et al., 1998; Mayou & Bryant, 2002; Smith et al., 2007; Australia: Jeavons, 2000). These studies varied in length of follow-up (4 months vs. 3 years) and in sample size (*n*<100 vs. *n*>500), and all used questionnaires, rather than the preferred diagnostic interviews, for assessing mental health. As such, RTC research with a minor injured cohort is yet to examine the long-term consequences of diagnosed psychiatric morbidity.

Wider research may also be relevant. Matsuoka et al. (2008) studied severely injured RTC survivors (*n*=100) in Japan and diagnosed a large minority (31%) of participants with psychiatric morbidity at 1 month post-RTC. A more general injury cohort (66% from RTC) in Australia (O'Donnell et al., 2013) found that psychiatric problems accounted for the most variance in disability at 1-year post-injury, over physical factors or pain severity. Of the available research, there are some reoccurring predictors of psychological or physical impairment, for example, PTSD, depression and mental illness diagnoses, perception of threat to life, self-reported disability, reported pain level, alcohol use, and injury severity. Other potential predictors without precedence include social support and litigation perceptions. Further, the interaction between physical and psychological problems has not been explored in a minor RTC sample. Clarifying our understanding of psychological morbidity and physical outcomes in the large, but under-studied population of RTC survivors with minor injuries is required.

Given the paucity of data on RTC survivors with minor injuries, the current study included participants who had not only sustained minor injuries but were also claimants within a common law fault-based Compulsory Third Party (CTP) scheme in Queensland, Australia. The population of Queensland is approximately 4.5 million (Australian Bureau of Statistics, 2012), and the Motor Accident Insurance Commission (MAIC) regulates and monitors the CTP scheme in the state. This scheme provides motor vehicle owners, drivers, passengers, and other injured persons with an insurance policy that covers their unlimited liability for personal injury caused by or in connection with the use of the insured motor vehicle in incidents to which the Motor Accident Insurance Act 1994 applies. Being a fault-based scheme, the injured party must be able to establish negligence against an owner or driver of a motor vehicle. The injured person then has the right to seek monetary compensation from the person at fault for their injury/losses in a court of law. Conversely, if an injured person was wholly at fault in the accident (i.e., there is no negligent party against whom a claim can be made) then the individual cannot obtain compensation.

There has been much controversy in the literature surrounding whether or not involvement in litigation/compensation predicts poorer health outcomes following injury (for reviews on this topic, see Blake et al., 1995; Carroll et al., 2011). A recent systematic review of predictors of PTSD for adult RTC survivors (Heron-Delaney, Kenardy, Charlton, & Matsuoka, 2013) indicated that involvement in litigation/the compensation process predicted development of PTSD, which is one possible health outcome that can be assessed following RTC. It seems plausible that involvement in litigation/compensation may increase the likelihood of developing PTSD due to the increased frequency of reminders of the RTC and the need to recount aspects of the trauma and continuing symptoms in what may be considered an unsupportive or stressful environment (i.e., with insurance managers or lawyers) (National Collaborating Centre for Mental Health, 2005). It is also possible that the influence of feelings of injustice and blame experienced by those not at fault is relevant in this sample, with previous research finding that those not at fault demonstrate more emotional and mental problems than those at fault (Littleton et al., 2012).

The primary aim of this study is to identify factors that can predict which individuals will experience long-term psychological or physical impairment following an RTC. The broad aims of the study are as follows: (1) describe the physical and mental health of compensable individuals who have sustained minor injuries in an RTC in Queensland; (2) assess perception of threat to life and objective injury severity and any relationship with mental health outcomes; (3) evaluate the role of mental health in determining outcomes for physical injury; (4) identify other factors (e.g., level of pain, general quality of life, social support, expectations regarding recovery) that can modify the course of recovery; (5) identify predictors of work absenteeism; (6) describe participant perceptions of the insurance claims process and explore relationships between these perceptions and physical functioning; (7) provide long-term follow-up on participants’ functioning at 24 months post-RTC; and (8) develop information that will facilitate early identification of individuals who may require expedited insurance claim settlement, and/or specialized attention/intervention. The unique element is the longitudinal study of a cohort of individuals suffering minor and moderate injuries from RTC.

This paper describes the design and measures in detail and the demographics of the cohort studied. Recruitment procedures and data collection methods are outlined, providing a background for future reports from this cohort.

## Method

### Participants

Participants were RTC survivors recruited from the MAIC records across an 18-month period between April 2009 and September 2010. To be on the MAIC database, individuals needed to have been involved in an RTC where the “at fault” vehicle was registered and had CTP insurance with a licensed Queensland CTP insurer. Thus, participants’ RTCs did not necessarily have to occur within Queensland; however, the vast majority did and similarly the majority of participants resided in Queensland. The study sample group does not represent the entire possible RTC cohort, as the participants are claimants in a common law fault-based scheme. Thus, at fault drivers (who are not compensable) are not included in this study, nor are individuals who may have been entitled to claim, but may not have lodged a claim for various reasons (e.g., may have had a minor injury that recovered).

### Eligibility criteria

The inclusion criteria were as follows: (1) RTC-related physical injury which was minor to serious as defined by the Abbreviated Injury Scale (AIS score of 1–3); individuals could be the driver/passenger of a car/motor bike, cyclist, or pedestrian involved in an RTC; (2) CTP claimants; (3) aged 18 years and older; (4) good English-speaking ability; (5) the RTC date must be within the last 3 months of the claim notification date; and (6) the claimant resides in Australia. The exclusion criteria were as follows: (1) cognitive impairment (subjectively assessed by the capacity to answer questions during the initial interview); (2) insufficient English language competence; and (3) a severe physical condition preventing the patient from tolerating the interview or completing the survey (e.g., stroke, paralysis). By using these inclusion and exclusion criteria, only people who had minor, moderate, or serious injuries (not severe or critical), as defined by the AIS, participated in the study.

### Procedure

Potential participants were identified from the MAIC's database and were sent a letter by MAIC inviting them to participate in the study (at approximately 3 months post-RTC). Accompanying the letter was the Participation Information Sheet (summary of the study), a consent form, and a reply-paid envelope to return their consent form. Once written informed consent had been obtained, the respondent was mailed the survey booklet together with instructions to return the completed survey in the enclosed, addressed reply-paid envelope. The surveys were estimated to take approximately 20–30 min to complete. Approximately 1 month after the survey had been mailed to the participant, staff external to the research team conducted a Computer-Assisted Telephone Interview (CATI). The phone interview lasted anywhere from 10 min to 1.5 hours, depending on the level of the participant's symptoms. The CATI staff were highly skilled in conducting phone interviews of this nature, and being independent from the research team, were unaware of all study aims and hypotheses. The CATI staff were specifically trained in the administration of the Composite International Diagnostic Interview (CIDI), with all eight interviewers demonstrating 100% agreement in an assessment of reliability. The same procedure of staggering survey booklet completion and phone interviews was implemented at Wave 2 and Wave 3. In general, Wave 1 assessment was completed 6 months post-RTC. Wave 2 assessment was completed approximately 12–15 months post-RTC and Wave 3 assessment was completed 24 months post-RTC. Participants were deemed not contactable for a given assessment/wave when all points of contact had been exhausted (i.e., home, work and mobile phone number of the study participant, and the contact details of a family member/close friend as an alternate contact). For telephone interviews, attempts to contact were made on five different occasions, on different days and at different times of day. For survey booklets, participants were reminded twice via phone to return their survey booklets if they had not been returned within 1 month of being posted to the participant. The Medical Research Educational Council Ethics Committee at the University of Queensland, Brisbane, approved this study.

### Measures

Measures were chosen on the basis of their established psychometric properties (see below for specific references) and their extensive use in previous research. With three exceptions, the measures listed below were used in the survey or interview at each of the three waves (Wave 1=6 months post-RTC, Wave 2=12–15 months post-RTC, and Wave 3=24 months post-RTC). Questions relating to return to work and claim status were only included at Wave 3; demographics were collected at Wave 1; questions relating to mental health history were asked at Wave 1 and Wave 2 only.

#### Survey booklet

The Örebro Musculoskeletal Pain Questionnaire (OMPQ) (Linton & Boersma, 2003) measures physical and functional level and adjustment to injury and pain. It screens for factors that may hamper recovery including emotional state, fear–avoidance beliefs and coping strategies. The OMPQ is a self-administered screening instrument containing 25 items, where all responses are indicated on a Likert scale or by ticking a box. A total score is calculated after inverting some items so that higher ratings always indicate higher levels of risk. In line with Linton & Boersma (2003), the OMPQ was divided into subscales of function, pain and absenteeism due to sick leave. To create the function scale score, items 17–21 (items relating to ability to participate in normal activities, e.g., weekly shopping) were summed, to provide a score ranging between 0 and 50. The pain scale score was derived by multiplying the intensity of pain rating by the frequency of pain rating, proving a score in the range of 0 to 100. For the psychometric properties of the OMPQ, see Hockings, McAuley, and Maher (2008) and Westman, Linton, Ohrvik, Wahlén, and Leppert (2008).

The Short Form 36v2 (SF-36v2; Ware, Kosinski, & Dewey, 2000) measures physical and mental health constructs as well as perceived health status and daily functioning. Respondents were instructed to describe their health in the past 4 weeks. The SF-36v2 consists of 36 items where participants indicate their response by selecting one option (from either three or five options) on a scale. Responses to the questions are then divided into eight sub-scales: physical functioning, role limitation because of physical functioning, bodily pain, general perception of health, vitality, social functioning, role limitation because of emotional functioning, and mental health. Scoring protocol from Ware et al. (2000) was utilized. The eight scales form two distinct higher-order clusters: physical health (first four subscales) and mental health (last four subscales). SF-36v2 items and scales are standardized to a 0–100 point scale, and higher scores indicate a better health state. The SF-36 has good psychometric properties (see Ware, Snow, Kosinski, & Gandek, 1993 for a review) and has been used extensively worldwide (Ware et al., 2000).

The Multidimensional Scale of Perceived Social Support (MSPSS; Zimet, Dahlem, Zimet, & Farley, 1988) is a 12-item self-report measure to assess perceptions of interpersonal functioning and social support. Each item is rated on a 1–7 Likert scale, with higher scores indicating greater levels of perceived support (e.g., I can talk about my problems with my friends). Three domains of social support can be scored: support from friends, family and a significant other. A global support score is also calculated which encompasses all three sub-scales. Several studies have confirmed a three-factor solution (corresponding to the three subscales) (Clara, Cox, Enns, Murray, & Torgrudc, 2003; Eker & Arkar, 1995; Kazarian & McCabe, 1991; Zimet, Powell, Farley, Werkman, & Berkoff, 1990), with the MSPSS showing good psychometric properties (Canty-Mitchell & Zimet, 2000; Clara et al., 2003; Zimet et al., 1988, 1990). The MSPSS has been used extensively among psychiatric patients and normal participants (e.g., Clara et al., 2003; Zimet et al., 1990).

The Impact of Event Scale Revised (IES-R; Weiss & Marmar, 1997) is a self-report measure, which was used to assess current subjective posttraumatic stress. The IES-R has 22 items and three subscales (avoidance, intrusion, and hyperarousal). Respondents rate their degree of distress for each item on a scale of 0 (not at all), 1 (a little bit), 2 (moderately), 3 (quite a bit), and 4 (extremely) with reference to the past 7 days. Scores on the IES-R range from 0 to 75, with higher scores indicating greater levels of posttraumatic stress. Items are scored according to the three subscales, and the subscale totals are summed to produce the total IES-R score. For the psychometric properties of the IES-R, see Brewin (2005) and Weiss and Marmar (1997).

The Hospital Anxiety and Depression Scale (HADS; Zigmond & Snaith, 1983) was used to assess depression and anxiety symptoms in the past week. The HADS is a self-report measure containing 14 items that are rated on a four-point Likert scale ranging from 0 to 3, with high scores denoting greater psychological distress. There are seven items in the subscale measuring anxiety and seven items in the subscale measuring depression, and the two scales are summed to give the total score. Possible scores range from 0 to 42 (with a maximum score of 21 for each subscale). The HADS has demonstrated good psychometric properties (for reviews see Bjelland, Dahl, Haug, & Neckelmann, 2002; Herrmann, 1997), and has been used extensively in a variety of populations, including hospital patients (e.g., Johnston, Pollard, & Hennessey, 2000) and the general population (e.g., Mykletun, Stordal, & Dahl, 2001).

The EQ-5D (The EuroQol Group, 1990) is a standardized measure of self-reported health status which relates to the respondent's situation at the time of completion. It provides a single index value for health status that can be used for economic appraisals. The EQ-5D includes five dimensions: mobility, self-care, usual activities, pain/discomfort and anxiety/depression. The respondent is asked to indicate their health state from one of three statements: no problems, some problems and severe problems. Each statement is assigned a one-digit number according to the level indicated: level 1 for no problem, level 2 indicates some problems and level 3 indicating extreme problems. The digit assigned to each dimension can be combined into a five-digit number describing the individual's unique health state (243 health states are possible if defined this way). For example, 11111 indicates no problems on any of the five dimensions. The EQ-5D health states can be converted into a single summary index by using a formula that attaches values (weights) to each of the levels in each dimension. The weights utilized are from Viney et al. (2011). The index is calculated by deducting the appropriate weights from 1, the value for full health. Each score represents a quality-adjusted life year (QALY) for each individual participant. A QALY is a measure which includes both the quality and the quantity of life lived. The QALY is based on the number of years of life that would be added if full health were restored. The EQ-5D is an internationally developed health/quality of life measure that has good psychometric properties (Brazier et al., 1993; Dyer et al., 2010) and has been used extensively throughout the world.

The Alcohol Use Disorders Identification Test (AUDIT) is a 10-item World Health Organization brief screening tool used to assess alcohol use, including hazardous and harmful alcohol use as well as alcohol dependence (Saunders, Aasland, Babor, De La Fuente, & Grant, 1993). The screen focuses on recent alcohol use. There are three domains: (1) hazardous alcohol use (frequency of drinking and typical quantity), (2) dependence symptoms (impaired control over drinking, increased salience of drinking and morning drinking), and (3) harmful alcohol use (guilt after drinking, blackouts, alcohol related injuries, others’ concern about drinking). Scores from each question range from 0 to 4, with the first response for each question scoring lowest and the last response scoring highest. Higher scores indicate greater and riskier alcohol use.

A recent systematic review of the literature has concluded that the AUDIT is the best screening tool for alcohol problems in primary care (Fiellin, Reid, & O'Connor, 2000). The AUDIT shows good psychometric properties across a number of populations and studies conducted throughout the world (Allen, Litten, Fertig, & Babor, 1997; Fleming, Barry, & Macdonald, 1991; Hays, Merz, & Nicholas, 1995).

Perceptions of CTP insurance scheme. A nine-item questionnaire was specifically designed for this study to measure participants’ perceptions of the CTP Insurance scheme and process. The questions assessed participants’ perceptions about their: (1) ability to understand the CTP claims process, (2) degree of involvement in the management of the claim, (3) degree of consultation, (4) degree of influence on the claims process, (5) expectation that they would recover from their injuries, (6) expectation that they would return to doing what they did before the injury, (7) belief in the fairness of the claims process, and (8) satisfaction during the claims process. A five-point Likert scale was utilized for each question. The final question asked participants to rate their health (both mental and physical) now, as compared with before the RTC, on a five-point Likert scale ranging from 1 (considerably worse) to 5 (as good as before or better).

Return to work and claim status. At Wave 3, participants completed a short additional, purpose-made, questionnaire about their work and claim status. Specifically, participants reported (1) whether or not their claim had been finalized, (2) whether they had returned to work in a full- or part-time capacity, and (3) if they were performing full or modified duties.

#### Computer assisted telephone interview

Demographics. Gender, age, education level, marital status and work status were collected from participants at Wave 1. Where participants did not complete the interview at Wave 1, this information was requested from MAIC.

Participants’ *mental health history* was assessed using the following questions: (1) “Have you even been to see a doctor or mental health professional about any psychological problems you had before your accident?”, (2) “Were you given a diagnosis?”, (3) “What was the diagnosis?”, (4) “Did you receive treatment for that problem?”.

Participants’ *perception of threat to life* was measured using the question “How much did you believe you were going to die during the accident?” The five-point scale ranged from 1 (not at all) to 5 (very strongly).

Disability and health were measured using the 12-item version of the World Health Organization Disability Assessment Schedule II (WHO-DAS-II; Ustün, Kostanjsek, Chatterji, & Rehm, 2010). Six domains are measured: cognition, mobility, self-care, getting along with others, life activities and participation in society. Items are rated on a five-point Likert scale from 1 (no difficulty) to 5 (extreme difficulty/inability to perform the activity). For each item that is positively endorsed, a follow-up question asks about the number of days (in the past 30 days) the participant has experienced the difficulty. For the psychometric properties of the WHO-DAS-II, see Ustün et al. (2010). For scoring purposes, the simple scoring method described in Ustün et al. (2010) was utilized, where the values for all questions are summed.

PTSD was assessed using the PTSD module from the Composite International Diagnostic Interview (CIDI-PTSD; Peters et al., 1996). The CIDI-PTSD interview is a structured diagnostic interview based on the *Diagnostic and Statistical Manual of Mental Disorders* (DSM-IV) criteria (American Psychiatric Association, 1994). This module consists of a series of screening questions, which are followed up by detailed questions about symptoms of psychiatric disorders. The questions elicit responses in a yes/no format, with skip patterns built in so that the next question answered is dependent upon the previous response given for some items. The CIDI is designed and validated for use by a trained lay interviewer to be administered as a computer-guided face-to-face or telephone interview. As such, the interview does not require the interviewer to exercise clinical judgment. The 12-month version was used. The CIDI has demonstrated good psychometric properties (Andrews & Peters, 1998; Breslau, Kessler, & Peterson, 1998; Quintana, Mari Jde, Ribeiro, Jorge, & Andreoli, 2012).

The CIDI Short Form (CIDI-SF; Kessler, Andrews, Mroczek, Ustun, & Wittchen, 1998) is series of short-form screening scales developed from the CIDI designed to be used by trained lay interviewers. A range of mental disorders are diagnosed according DSM-IV criteria (American Psychiatric Association, 1994). Each of the CIDI-SF sections include a series of screening questions followed by detailed questions based on DSM-IV criteria. Like the CIDI, yes/no format is utilized with built in skip patterns, thus the interview does not require the interviewer to exercise clinical judgment. The 12-month version was used. The specific sections utilized were major depressive episode, generalized anxiety disorder, agoraphobia, and panic attack. The final two modules allowed for a diagnosis of agoraphobia with and without panic disorder and panic attacks with and without agoraphobia. Overall, a strong relationship exists between diagnoses based on the CIDI-SF and the full CIDI (Kessler et al., 1998).

Health care utilization. Two questions were devised which related to the level of contact participants had with medical doctors/other health professionals since their accident for a physical injury or other problem; the first asked for the number of health professional visits relating to the RTC, and second asked for the number which were not related to the RTC. If a patient saw the same health professional/practice more than once in a given day, this was only counted as one visit.

#### Injury list

A list of injuries coded using the 2005 version of the AIS was provided by MAIC for each participant. This enabled the calculation of an injury severity score (ISS). The ISS is a measure of injury severity that provides an overall score for patients with multiple injuries (Baker, O'Neill, Haddon, & Long, 1974). Each injury is assigned an AIS score and is classified according to one of six body regions (head, face, chest, abdomen, extremities, or external) (Association of the Advancement of Automotive Medicine, 2005). AIS scores range from 1 to 6 (six represents a fatal injury). The highest AIS score in each body region is used to calculate the ISS. The three body regions with the highest AIS scores (i.e., most severe injuries) have their score squared and added together to produce the ISS. ISS scores range from 0 to 75. Many different injury patterns can result in the same ISS. Similarly, people with injuries with the same severity rating can experience different functional impacts. The following classification system for ISS was utilized: 1–3=minor injury (consistent with Mayou & Bryant, 2002), 4–8=moderate injury, nine or more=serious injury. This system is in line with AIS coding, such that those with an ISS of 1–3 necessarily only have a maximum AIS score of 1, which is classified as minor according to the AIS coding system (Association of the Advancement of Automotive Medicine, 2005). Similarly, those with an ISS of 4–8 have a maximum AIS score of 2. This classification system is in line with Copes et al. (1988).

## Results

**Fig 1:**
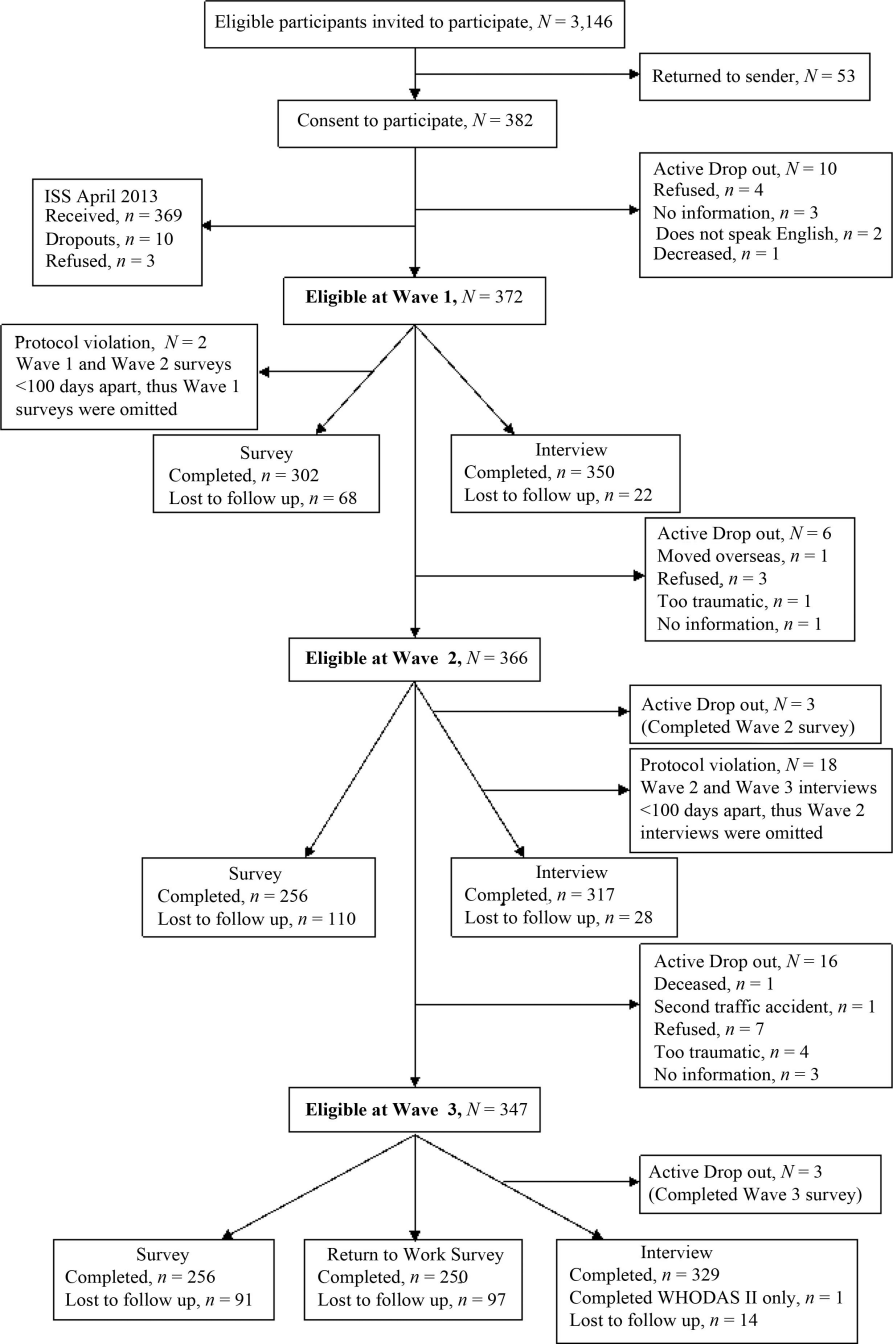
Flow chart of number of participants and drop-outs for each measure at each wave.

Of the 3,146 eligible people invited to participate in the study, 382 (12%) consented. Of these 382 participants, the lowest number of responses throughout the study was for the Return to Work Survey at Wave 3 (*n*=250), equating to a retention of participants of at least 65% at each wave. See Fig. 1 for a flow chart of recruitment and sample sizes at each wave. Thirty-two participants withdrew from the study due to explicit refusal to participate when the interviewer initially made contact, insufficient English to participate, relocation overseas, reported they found the interview process too traumatic, involvement in a second RTC or death (see Fig. 1). Other participants did not complete the interview and/or survey at Wave 1 because interviewers were unable to make contact with these participants after numerous attempts using alternate forms of contact. These participants were lost to follow up. For two participants, their Wave 1 and Wave 2 surveys were conducted less than 100 days apart, with the Wave 1 survey being completed very late while the Wave 2 survey was completed on time. For these two participants, Wave 1 data were deleted. Similarly, 18 participants had their Wave 2 and Wave 3 interviews completed less than 100 days apart, therefore their Wave 2 data were not retained.

Mean times between RTC and both survey and interview completion for each wave are reported in Table 1. The mean age of the sample at Wave 1 (*n*=350) was 48.6 years (* SD*=14.90; range=19–94 years), with only 2.7% (*n*=10) of the sample being older than 75 years. The median ISS for the sample was 3 (IQR=1–5; range=1–24), with 65% (*n*=225) of individuals sustaining minor injury. Other Wave 1 sample characteristics are presented in Table 2. A comparison of individuals who participated at Wave 1 and those who declined participation (*n*=2,723) showed that those who participated were significantly older that those who declined (*M*=39.7, *SD*=14.5, *t*_(438)_=10.55, *p*<.001), and had a lower proportion of minor injury than those who declined (82%, *χ* ^2^=58.75, *p*<.001). There was no significant difference in the percentage of females between participants (62%) and non-participants (57%).

**Table 1:** Mean time (in months) between RTC and survey/interview completion for each wave

	Survey completion		Interview completion	
		
Wave	Mean (SD)	Range	Mean (SD)	Range
1	5.14 (1.12)	2.80–10.03	6.67 (1.29)	3.47–11.50
2	12.16 (1.51)	10.1–21.70	15.06 (2.40)	10.97–22.97
3	23.79 (1.15)	21.97–29.07	24.73 (1.33)	22.87–34.90

**Table 2:** Wave 1 sample characteristics based on participants who completed an interview (*N*=350)

	Mean (SD)	Range	*N*	%
Age	48.62 (14.90)	19–94		
Gender				
Male			133	38
Female			217	62
Education (years)	14.85 (3.87)	0–30		
Marital status				
Never married			60	17
Currently married			200	57
Separated			11	3
Divorced			39	11
Widowed			16	5
Cohabiting			24	7
Employment status				
Paid work			194	55
Self employed			30	9
Non paid work			2	1
Student			12	3
Homemaker			16	5
Retired			42	12
Unemployed (health reasons)			34	10
Unemployed (other reasons)			11	3
Other			9	3
Road user type involved in RTC				
Driver			222	63
Passenger			58	17
Pedestrian			21	6
Cyclist			51	15
Fatality				
Yes			7	2
No			343	98
Injury severity score (*n* = 347)				
Minor (1–3)			225	65
Moderate (4–8)			81	23
Serious (≥9)			41	12

## Discussion

This study provides the first comprehensive, long-term (2-year) investigation specifically focusing on an RTC sample with largely minor injuries who are claimants in a common law fault-based personal injury scheme. To date, there are only three other studies which investigate outcomes in minor injury RTC samples (Jeavons, 2000; Mayou & Bryant, 2002; Smith et al., 2007). Given the large number of RTCs worldwide each year and the associated cost, and the large proportion of RTCs which involve minor/moderate injuries, it is important to understand the consequences and attempt to identify predictors of which individuals are less likely to recover. This study has a strong methodology. It utilizes a multitude of measures to assess a broad range of constructs, and has the advantage of using a structured clinical interview to assess psychological outcomes rather than a self-report screen.

This study will make an important contribution to our understanding of disability, physical functioning, pain and mental health following an RTC, and provide indicators for early identification of those at risk of developing physical and psychological disorders following an RTC where the injuries sustained are minor/moderate. In future analyses using this protocol, the interaction between physical and mental health outcomes will be a specific focus, as it is becoming increasingly apparent that this is vital in predicting recovery (Sterling & Kenardy, 2011). Future analyses will also examine predictors of failure to return to work, providing information for influencing policy and practice in injury management and post-injury rehabilitation.

This study focuses on an exclusively RTC sample. The focus on RTC-related trauma follows from concerns that unique problems may be associated with specific types of traumatic injury. For example, traumatic events that are characterized as involving intention to injure (assaults) are distinct from those with no intent. Furthermore, there is a wide range of different types of unintentional injuries (e.g., work-related injuries, accidents occurring in the home). It is possible that different types of unintentional trauma involve distinct factors which may influence psychological symptomology. For example, injuries sustained in the home are unlikely to result in litigation and compensation processes, whereas RTC-related trauma frequently involve negligence and results in litigation and/or compensation processes. Research has shown that the compensation process is associated with on-going symptomology (Carroll et al., 2011), which is important to note in the current RTC-related sample. Whilst generalizability may be constrained, this approach provides greater precision in linking trauma and circumstances of trauma to outcome.

### Limitations

A potential limitation of the study is the relatively low participation rate, which is likely to be a consequence of consent obtained via post rather than in person. It is difficult to recruit minor injury RTC samples because the individuals are not hospitalized and thus no direct contact can be made with potential participants. Other studies of RTC survivors have reported higher participation rates; however, recruitment involved personal contact (Matsuoka et al., 2008). Previous studies that have recruited participants via post report similar participation rates (e.g., Smith et al., 2007). In addition to the relatively low participation rate, participants who consented were found to be older and more severely injured than those who declined to participate; there were no gender differences. This finding may also be symptomatic of recruiting from the minor injury cohort, where those with very minor injuries may have recovered prior to being contacted regarding participation in the study (approximately 3 months post-RTC). These factors may affect the generalizability of the findings, and future reported prevalence estimates should be interpreted with caution.

Finally, the sample group does not represent the entire possible RTC cohort. At-fault drivers (who are not compensable) were not included in this study; only individuals who could establish that they were not at fault in the RTC were included. The influence of feelings of injustice and blame experienced by those not at fault needs to be acknowledged in future analyses, with previous research finding that those not at fault demonstrate more emotional and mental problems than those at fault (Littleton et al., 2012).

## Conclusions

The results from this study will provide detailed information on physical factors/outcomes (disability, functioning, pain, injury severity), psychological factors/outcomes (PTSD, major depressive episode, generalized anxiety disorder, agoraphobia, panic attack, quality of life, perception of threat to life during the RTC, expectations regarding recovery), social support, alcohol use, claim status, health care utilization and return to work for RTC survivors who have sustained minor/moderate injuries. Analysis of this information will inform policy and practice in injury management and post-injury rehabilitation.
